# Ultra-Rapid Freezing Preserves Morphofunctional Integrity and Fertilizing Ability of Epididymal Cat Spermatozoa

**DOI:** 10.3389/fvets.2022.866953

**Published:** 2022-06-14

**Authors:** Martina Colombo, Maria Giorgia Morselli, Jennifer Zahmel, Gaia Cecilia Luvoni

**Affiliations:** ^1^Dipartimento di Medicina Veterinaria e Scienze Animali (DIVAS), Università degli Studi di Milano, Milan, Italy; ^2^Department of Reproduction Biology, Leibniz-Institute for Zoo and Wildlife Research, Berlin, Germany

**Keywords:** cryopreservation, embryo, feline, field, ICSI, pellet, straw, vitrification

## Abstract

Vitrification and ultra-rapid freezing, which are more commonly used for oocytes and embryos, have recently been applied to spermatozoa in an attempt to make semen cryopreservation in field conditions easier compared to conventional freezing. It is well-known that in case of unexpected death of rare and wild animals, preserving epididymal spermatozoa from isolated testicles represents a great chance of salvaging male germplasm for future use in assisted reproductive technologies. The aim of this study was to evaluate the morphofunctional integrity of cat epididymal spermatozoa ultra-rapid frozen in pellets or straws with two different extenders [E1 (Tris buffer with 20% egg yolk and 0.25 M sucrose) or E2 (Ham's F10 with 1% bovine serum albumin and 0.4 M sucrose)] and to test whether spermatozoa preserved by the best combination were able to fertilize oocytes and produce embryos *in vitro* by intracytoplasmic sperm injection (ICSI) of *in vitro* matured cat oocytes. The results showed that E1 and E2 in straw or pellet were comparable (at warming, about 30% normal morphology, 45% intact membranes, and 20% intact acrosomes), except for post-warming motility that was better maintained along time by E1 pellet (21.7 ± 7.4% at warming and 3.6 ± 2.9% after 6 h). Such spermatozoa could fertilize conspecific oocytes and support embryonic development (cleavage 35.5%) as well as frozen control spermatozoa (cleavage 54.29%, *p* = 0.22). In conclusion, cat epididymal spermatozoa better maintained their morphofunctional features after ultra-rapid freezing with E1 and could successfully produce embryos *in vitro* after ICSI. This underscores their usefulness as cryobanked material for fertility and biodiversity preservation purposes.

## Introduction

The current standard cryopreservation method for spermatozoa is slow-freezing (1), but vitrification or ultra-rapid freezing could offer some logistical advantages; therefore, they are being investigated as cryopreservation methods in several animal species and in humans (2–6). They are faster and easier than slow-freezing, they require less equipment, and they can usually be performed by less trained personnel. In addition, their results were often comparable to those of conventional slow-freezing, concerning motility, membrane, and/or acrosome integrity in donkeys (7) and cats (8), or they were even superior for the same parameters in horses (9) or for motility and morphology in humans (10, 11). Most importantly, vitrified spermatozoa already led to several live births in humans (12–16). Therefore, spermatozoa vitrification or ultra-rapid freezing could be used as alternative and cost-effective techniques for long-term male fertility preservation, especially in field conditions or in zoos, for instance, in case of a sudden death of rare or endangered animals.

However, the choice of vitrification could also represent a risk for spermatozoa cryopreservation, especially if considering the amount of cryoprotectants that might be necessary to create a viscous solution. Indeed, cryoprotectants might be toxic (17), especially at higher temperatures (18), and they could cause osmotic stress (19), but modern vitrification approaches try to reduce or avoid their use. Compared to other cell types, it seems that spermatozoa are less tolerant to permeant cryoprotectants (19). Instead, the use of non-permeant cryoprotectants, such as proteins or sugars, could be advantageous thanks to their reduced cytotoxicity and their osmo-protective properties (20, 21). Thus, the current trend is to attempt spermatozoa vitrification without cryoprotectants at all or with the addition of a small amount of non-permeant cryoprotectants, mainly sucrose and albumin (22).

Although most work on the development of the technique and optimization of cryoprotectants has probably been done in the human species, vitrification, as well as ultra-rapid freezing, is also of high interest for animals and biomedical models, such as the domestic cat. The cat is a good model for related wild endangered feline species, for which the development of easy, diffusible, and field-feasible germplasm cryopreservation techniques is urgent. In the domestic cat, field-feasible preservation of epididymal spermatozoa (23) and of urethral catheterization-derived ejaculated semen (8) have been attempted. In epididymal spermatozoa, motility was severely affected compared to fresh samples (23), whereas motility and acrosome integrity of ejaculated vitrified spermatozoa, as well as IVF success, were comparable to those of conventionally frozen controls (8). Most notably, the birth of kittens was also obtained (24).

Thus, cat spermatozoa field-feasible preservation seems promising, but it still needs consideration, especially if the samples are of suboptimal quality, such as those that are often collected from wild felids after their sudden death from isolated epididymides. A total of two of the biggest issues are oligozoospermia, especially in small cats (25), and teratozoospermia, a recognized common feature of felid semen (26). In addition, seasonality also plays a role in semen quality (27, 28). For such samples, it is unlikely to have a sufficient number of motile spermatozoa for artificial insemination or even IVF, and the only option to use these gametes for embryo production is the intracytoplasmic sperm injection (ICSI).

However, the optimization of conditions of the cryopreservation step is still needed. For what concerns the extender, following the determination of the most suitable sucrose concentration (23), the addition of other protective compounds could be beneficial. For instance, the addition of a membrane-protecting agent, such as egg yolk, should be evaluated, since its non-animal counterpart soy lecithin was already employed successfully (8). In addition, instead of using simple Dulbecco's phosphate-buffered saline (23), the use of richer media could be advantageous to better support spermatozoa metabolism. On the other hand, for what concerns the cryo-carriers, several supports developed for the vitrification of other cells (e.g., oocytes) can be employed, and some devices were designed specifically for spermatozoa. However, cost, ease of use, and field applicability should be considered, and semen straws (23) or pellets (8) are probably the most convenient options, especially when thinking about gamete rescue far from an equipped laboratory.

The conditions encountered during spermatozoa cryopreservation in wild felids (e.g., single specimen, poor semen quality, and quantity) prompted the implementation of this study. Ultra-rapid freezing was applied to exploit the convenience, in terms of time, of direct immersion of samples into liquid nitrogen and the easy handling of a relatively reduced sample volume. The aim was to explore the effects of different ultra-rapid freezing extenders and systems on cat spermatozoa morphofunctional features and fertilizing ability as a model for wild endangered felid. In particular, based on the previous reports, it was hereby assessed (1) the morphofunctional integrity of spermatozoa ultra-rapid frozen with two extenders and two systems (straw and pellet) and (2) the fertilizing ability following ICSI of spermatozoa ultra-rapid frozen with the best combination of extender and system.

## Materials and Methods

### Chemicals and Reagents

All chemicals and reagents were purchased from Merck KGaA (Darmstadt, Germany), unless otherwise stated.

### Experimental Design

In Experiment I, epididymal spermatozoa collected from the isolated gonads of domestic cats (*Felis catus, n* = 10) were ultra-rapid frozen. The collected material from each individual (both the gonads) was treated separately (i.e., without sample pooling), as it would happen for the gamete rescue of wild or rare animals, after unexpected death in the field. Spermatozoa quality (motility, morphology, membrane, and acrosome integrity) before ultra-rapid freezing (fresh control) and after ultra-rapid freezing with two different extenders (E1 and E2) and two systems (straw and pellet) was assessed. In ultra-rapid frozen samples, assessments were performed at warming (T0), 3 h post-warming (T3), and 6 h post-warming (T6). In Experiment II, the best combination of extender and system was chosen to assess the fertilizing ability of ultra-rapid frozen spermatozoa by ICSI of conspecific *in vitro* matured oocytes, compared to that of conventionally slow-frozen cat epididymal spermatozoa (positive control). Sham-ICSI was used as a control for parthenogenetic activation.

### Collection of Epididymal Spermatozoa (Experiment I)

Feline epididymal spermatozoa were obtained after routine orchiectomy. The epididymides were dissected from isolated testicles and placed in a Petri dish containing the recovery medium [2 mM L-glutamine and 5% fetal bovine serum (FBS) in Ham's F-10 (HF-10) medium]. Spermatozoa were obtained from *vas deferens* and *cauda epididymis* by direct squeezing in the recovery medium under a stereomicroscope, collected with a pipette, and placed in a test tube to be used for the experiments. Spermatozoa from the two gonads of each cat were pooled together.

### Ultra-Rapid Freezing and Warming of Epididymal Spermatozoa (Experiments I and II)

Ultra-rapid freezing and warming were performed as a modification of the protocols previously described for the domestic cat (8, 23) and dog (29). A total of two extenders, E1 [Tris buffer (3.025 g Tris-hydroxymethylaminomethane, 1.7 g citric acid, and 1.25 g fructose in 100 ml distilled water, (30)) with 20% egg yolk and 0.25 M sucrose] and E2 [Ham's F10 with 1% bovine serum albumin (BSA) and 0.4 M sucrose] and two systems (straw and pellet) were used in combination, obtaining four experimental groups (E1 straw, E1 pellet, E2 straw, and E2 pellet). Spermatozoa recovered from each cat were divided into two extenders, and half of the spermatozoa in each extender was ultra-rapid frozen in pellets and half in straws. In particular, the ultra-rapid freezing procedure included the addition of extender (E1 or E2) at 1:1 (v:v) ratio to the spermatozoa and their packing as follows:

-Pellet: after 5 min of equilibration, 10 μl of semen was pipetted directly into an open container of liquid nitrogen (8) from a height of about 10 cm (29). Then, pellets were moved with a cold clamp in a labeled cryotube and placed in a liquid nitrogen tank for storage.
- Straw: 10 μl of semen was loaded in 0.25-ml straws, which were exposed for 2 min at nitrogen vapors (on a floating styrofoam platform, 2 cm above the liquid nitrogen surface) and then plunged into liquid nitrogen as in (23). Straws were then moved to the tank for storage.

The warming procedure differed depending on the system and extender. The warming media, kept in a water bath at 37°C, were Tris buffer for spermatozoa preserved with E1, and HF-10 for spermatozoa preserved in E2. Straw-stored spermatozoa were released in 300 μl of warming medium cutting the straw, whereas pellets were directly moved to a test tube containing the same amount of warming medium. After warming, spermatozoa were centrifuged (300 g × 8 min) and resuspended in 100 μl of fresh warming medium after supernatant removal.

For Experiment I, for each cat, 6 E1 pellets, 6 E2 pellets, 6 E1 straws, and 6 E2 straws were warmed at the same time for sperm quality evaluation. For Experiment II, one E1 pellet was warmed for each ICSI replicate; two different cats were alternated between different ICSIs.

### Sperm Quality Evaluation (Experiment I)

Evaluations of motility, morphology, membrane integrity, and acrosome status have been performed on all the groups (fresh and ultra-rapid frozen) as follows. For each sample and for each analysis, 100 spermatozoa were evaluated.

#### Motility

In fresh samples, the percentage of motile spermatozoa was evaluated subjectively under an optical microscope (Axiovert 100, Zeiss, Italy), whereas in cryopreserved samples, few spermatozoa with a progressive motility were individually counted.

#### Morphology

Spermatozoa were smeared on slides and stained with Bengal Rose and Victoria Blue B. Spermatozoa were evaluated under a light microscope with an oil immersion objective at 100 × magnification. Normal and abnormal spermatozoa (with alterations in head, midpiece, and tail) were recorded. Defects were classified, according to their origin and severity, in major and minor as in (31). Briefly, major defects were included, for instance, the alterations in head shape, double heads, midpiece alterations, and double tails, whereas minor were included, for instance, detached heads, distal droplets, and bent tails.

#### Membrane Integrity

Membrane integrity was assessed by the hypo-osmotic swelling test (HOS), as described for canine semen (32, 33). Briefly, an aliquot of semen (5 μl) was incubated in a pre-heated 150 mOsm hypotonic solution (0.735 g sodium citrate and 1.351 g fructose in 100 ml distilled water) at 37°C and 5% CO_2_ for 30 min. Spermatozoa were evaluated under a light microscope with oil immersion and objective at 100 × magnification and classified as curly (intact membranes) or not curly (damaged membranes).

#### Acrosome Integrity

Acrosome status was evaluated by the peanut agglutinin (PNA) conjugated with fluorescein isothiocyanate (FITC) and propidium iodide (PI) staining, under a fluorescent microscope according to the procedure previously described (34, 35). Acrosomes of stained spermatozoa were classified as intact (spermatozoa displaying intensively bright green fluorescence of the acrosomal cap, indicating an intact outer acrosomal membrane) or damaged (spermatozoa displaying disrupted fluorescence, fluorescent band at the equatorial segment, or no green fluorescence, indicating damages to the outer acrosomal membrane).

### *In vitro* Embryo Production (Experiment II)

#### Oocyte Collection and *in vitro* Maturation

Cumulus oocyte complexes (COCs) were recovered from isolated cat ovaries (*n* = 26) following routine ovariectomies. After surgery, ovaries were immediately placed in PBS with a mixture of antibiotics (AB) and antimycotics (100 IU/ml of penicillin G sodium, 0.1 mg/ml of streptomycin sulfate, 0.25 μg/ml of amphotericin B) and transported to the laboratory at room temperature (RT). Cat COCs were obtained by mincing the ovaries with a scalpel blade in PBS and AB with 0.1% (w/v) polyvinyl alcohol (PVA). Only grade I oocytes [completely surrounded by more than five layers of compacted cumulus cells and with a homogeneous, dark ooplasm; (36)] were selected (*n* = 141). Oocytes were *in vitro* matured for 24 h in a controlled atmosphere (38.5°C and 5% CO_2_ in air) in 100 μl microdrops of maturation medium [medium 199 supplemented with 3 mg/ml of BSA, 10 ng/ml of epidermal growth factor (EGF), 0.6 mM cysteine, and 0.5 IU/ml follicle-stimulating hormone + 0.5 IU/ml luteinizing hormone (Pluset®, Calier, Spain)] (37) covered by mineral oil in Petri dishes. At the end of IVM, oocytes were observed under the microscope to assess cumulus expansion and they were prepared for ICSI.

#### Conventional Slow-Freezing of Cat Epididymal Spermatozoa (Control Spermatozoa)

Spermatozoa to be used as positive control for ICSI were frozen according to the Uppsala method originally developed for canine semen (30), using egg yolk-Tris-based extenders containing Equex, which was also tested in cat epididymal spermatozoa (38). Spermatozoa from one cat were used. Briefly, after the collection by squeezing from isolated epididymides as described above, spermatozoa were centrifuged (700 g × 5 min). The supernatant was removed and spermatozoa were diluted at room temperature with the first freezing extender. After cooling at 4°C for 3–4 h, the second freezing extender was added [overall composition of freezing extender: Tris buffer with final concentrations of 5% glycerol, 1% Equex STM (Nova Chemical Sales Inc., Scituate, MA, USA) and 20% egg yolk (30)], and spermatozoa suspension was loaded into 0.25-ml straws, which were frozen in liquid nitrogen vapors before plunging into the liquid and storage.

For thawing, straws were exposed to air for 10 s and then immersed in a water bath at 37°C for 30 s. After retrieval, spermatozoa were centrifuged (700 g x 5 min) and resuspended in Tris buffer after supernatant removal.

#### Intracytoplasmic Sperm Injection and Embryo *in vitro* Culture

An inverted microscope (Axiovert 100) coupled to a micromanipulator system (CellTram® 4r, Eppendorf, Italy) was used for the Intracytoplasmic Sperm Injection (ICSI) procedures. At the end of IVM, oocytes were deprived of cumulus cells by mechanical displacement with a *Stripper* pipette (BioTipp, Waterford, Ireland) equipped with a 125-μm-diameter tip to allow for the visualization of the polar body as a sign of maturation. Available mature oocytes in each replicate were divided into three groups: (1) oocytes to be injected with slow-frozen spermatozoa (positive control); (2) oocytes to be injected with E1 pellet ultra-rapid frozen spermatozoa (treatment); and (3) oocytes for sham-injection (negative control). Denuded oocytes were moved to 10-μl drops of ICSI medium (medium 199 supplemented with 3 mg/ml of BSA, 0.6 Mm di-cysteine, and 12.5 mM HEPES), one oocyte in each drop, in a Petri dish. Spermatozoa (ultra-rapid frozen and slow-frozen) were placed in 10-μl drops of 12% (w/v) polyvinylpyrrolidone (PVP, Sage IVF Inc., Cooper Surgical, Malov, Denmark). Drops were covered with mineral oil, and the dish was placed on the heated stage of the microscope for the ICSI. Frozen spermatozoa, used as positive control, were of proven *in vitro* fertility (i.e., capable of producing embryos *in vitro* by standard IVF).

The microinjection was performed as previously described (39). Briefly, motile spermatozoa without apparent morphological abnormalities were immobilized by drawing the injection pipette (Transfertips-F, Eppendorf) across the mid-piece, and then aspirated, tail first, into the injection pipette. Each spermatozoon was injected carefully into a mature oocyte from a 3 o'clock position, while the oocyte polar body was placed on a 6 or 12 o'clock position to avoid damage to the metaphase II spindle. To calculate the rate of parthenogenetic activation, a group of oocytes was used for sham-ICSI, where the procedure was performed in the same way described above, but without a spermatozoon.

Injected oocytes were washed two times in embryo *in vitro* culture (IVC) medium [HF-10 supplemented with 5% FBS, 0.11 mg/ml sodium pyruvate, 0.075 mg/ml L-glutamine, and 0.06 mg/ml gentamicin (40)] and then placed in a 4-well dish in 500 μL IVC medium covered by mineral oil (one well for each experimental group, 4–10 presumptive embryos in each well). The dish was placed in a controlled atmosphere (38.5°C and 5% CO_2_ in air) for seven days for embryo culture. Cleavage was assessed 48 h after ICSI (day two), and uncleaved or degenerating oocytes were excluded from culture and fixed (i.e., air-dried on a slide and fixed in 80% ethanol overnight at 4°C). Progression of embryo development was evaluated on days three and five. Arrested or degenerating embryos, as well as all remaining embryos on day seven, were fixed.

#### Fertilization Assessment (Evaluation of Nuclear Status)

All fixed oocytes and embryos were evaluated to confirm their nuclear configuration (oocytes) or the number of blastomeres (embryos) by a bis-benzimide (Hoechst 33342) staining. Briefly, oocytes or embryos on a slide with a minimum amount of medium were covered by 10 μl of Hoechst working solution (0.01 mg/ml). After 5 min of incubation in the dark, the Hoechst solution was removed and the slides were covered with antifading solution and a coverslip. Finally, stained oocytes and embryos were observed under a fluorescent microscope with 40X objective.

**Figure 1:**
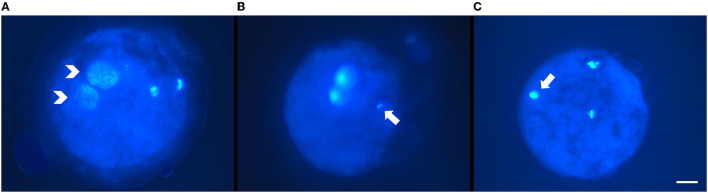
Representative micrographs of fertilization pattern obtained after intracytoplasmic sperm injection (ICSI) of ultra-rapid frozen or control slow-frozen feline spermatozoa and Hoechst staining. **(A)** Pattern of normal fertilization: two pronuclei (white arrowheads). **(B,C)** Other fertilization patterns: **(B)** metaphase II arrest with intact sperm head (white arrow); metaphase II was placed out of focus to allow better visualization of the sperm head. **(C)** metaphase II arrest with decondensing sperm head (white arrow). Scale bar: 20 μm, objective 40X.

Nuclear configurations were classified as follows (41):

- Normal fertilization: one (female) pronucleus with decondensed sperm head, two pronuclei (Figure 1A), or cleaved embryos with the extrusion of the second polar body;
- Other patterns of fertilization: oocyte activation with intact sperm head, metaphase II (MII) arrest with intact sperm head (Figure 1B), and MII arrest with decondensed sperm head (Figure 1C);
- Degenerated oocytes: dispersed chromatin within the ooplasm or unrecognizable patterns.
- For later-stage embryos, the number of blastomeres nuclei was counted to confirm the developmental stage.

### Statistical Analysis (Experiments I and II)

In Experiment I, data are reported as mean ± SD. Factors related to ultra-rapid freezing (extender, system, and post-warming time) were combined obtaining 12 groups for cryopreserved spermatozoa, which were compared to fresh controls. Data were tested for homogeneity of variance using Levene's test and for normality using Shapiro–Wilk test. According to this, they were analyzed by one-way ANOVA (membrane integrity) followed by a Tukey's *post hoc* test or by Kruskal–Wallis non-parametric one-way ANOVA (motility, morphology, acrosome integrity) followed by Dwass–Steel–Critchlow–Fligner pairwise comparisons. In Experiment II, data are reported as percentages, and they were analyzed by Fisher's exact test. Significance was set at *p* ≤ 0.05.

## Results

### Experiment I

**Figure 2:**
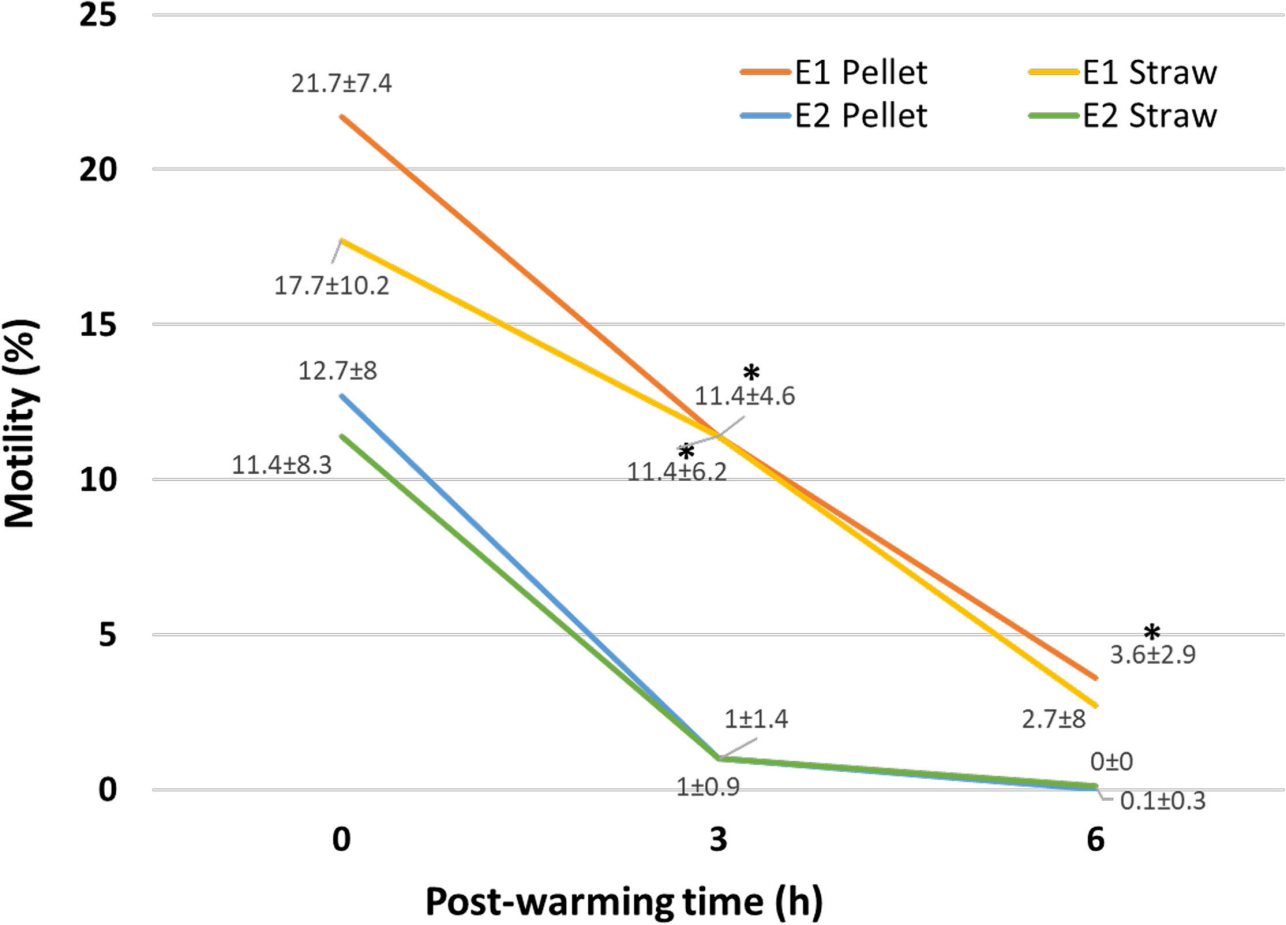
Motility of ultra-rapid frozen cat epididymal spermatozoa after warming (0 h) and 3 h (3 h) and 6 h (6 h) post-warming. Ultra-rapid freezing was performed with different extenders (E1, E2) and systems (pellet, straw). Within the same time point, *on E1, data indicate significant different values (*p* < 0.05) vs. E2.

Results of motility, morphology, membrane, and acrosome integrity of fresh and ultra-rapid frozen cat epididymal spermatozoa are shown in Figure 2 and Table 1.

**Table 1:** Morphology, membrane, and acrosome integrity of cat epididymal spermatozoa after collection (fresh) or after ultra-rapid freezing (ultra-rapid frozen) with different extenders (E1, E2) and systems (pellet, straw).

**Treatment**	**Extender**	**System**	**Post-warming time (h)**	**Normal morphology %**	**Abnormal morphology**		**Intact membranes %**	**Intact acrosomes %**
					**Major defects %**	**Minor defects %**		
Fresh	/	/	/	50.9 ± 21.4^ab^	23.9 ± 14.2	25.1 ± 10.7^ab^	75.3 ± 13.9^a^	78.2 ± 9.7^a^
Ultra-rapid frozen	E1	Pellet	0	32.4 ± 16.4^bc^	35 ± 20.6	32.5 ± 10.4^bc^	46.2 ± 12.7^b^	25.7 ± 23.2^bc^
			3	25.4 ± 20.3^bc^	34.3 ± 17.7	40.3 ± 17.6^bc^	38.3 ± 10.8^b^	10.1 ± 9^bc^
			6	25.7 ± 11.6^bc^	37.4 ± 10.1	36.9 ± 11.2^bc^	32.2 ± 9.1^b^	10.5 ± 15.6^bc^
	E1	Straw	0	29.7 ± 16.7^bc^	31.9 ± 16.7	38.4 ± 5.4^bc^	50.2 ± 14.3^b^	15.7 ± 13.2^bc^
			3	30 ± 21.8^bc^	36.5 ± 26.5	33.5 ± 17.3^bc^	40.1 ± 13^b^	9.65 ± 10.2^bc^
			6	27.6 ± 24.8^bc^	23.3 ± 19.6	49.2 ± 21.2^bc^	39.2 ± 10.6^b^	6.99 ± 9.1^bc^
	E2	Pellet	0	29.9 ± 21.2^bc^	40.6 ± 18.6	29.5 ± 5.9^ab^	45.9 ± 16.2^b^	19.8 ± 13.5^b^
			3	22 ± 14.3^bc^	45.3 ± 21	32.7 ± 21.5^bc^	41.6 ± 11^b^	8.97 ± 8^bc^
			6	12.9 ± 18.8^bc^	38.7 ± 20.5	48.4 ± 21^bc^	37.2 ± 10.8^b^	3.16 ± 4.9^c^
	E2	Straw	0	25 ± 19.6^bc^	37.9 ± 22.5	37.1 ± 9.5^bc^	46.3 ± 16.4^b^	16.8 ± 13^bc^
			3	17.5 ± 13.1^c^	31.9 ± 16.3	50.6 ± 11.8^c^	37.5 ± 12.5^b^	8.9 ± 7.2^bc^
			6	19.5 ± 20.2^bc^	33.3 ± 17.8	47.2 ± 17.2^bc^	34.5 ± 10.2^b^	6.31 ± 7.2^bc^

^a, b, c^*Different superscripts indicate significant differences within columns (p ≤ 0.05)*.

As expected, fresh spermatozoa had better parameters than ultra-rapid frozen ones. Sperm motility decreased (*p* = 0.01) in all the ultra-rapid frozen groups compared to fresh samples (motility of fresh control: 61 ± 15.2%). It was similar between ultra-rapid frozen groups at warming (*p* = 1), whereas, regardless of the ultra-rapid freezing system, motility at 3 h post-warming differed between E1 and E2 (*p* = 0.018), being higher in E1, without difference between pellet and straw (*p* = 1). Motility at 6 h post-warming was higher in E1 pellet than in E2 pellet (*p* = 0.012) and E2 straw (*p* = 0.025). It seemed that motility after warming was better maintained in E1 samples, since motility at 6 h post-warming was 0% in 9 out of 10 cats for E2, whereas E1 pellet maintained some motile spermatozoa in 9 out of 10 cats and E1 straw did the same in 7 out of 10.

Concerning morphology, normal spermatozoa tended to decrease after ultra-rapid freezing, even if with few significant differences (fresh vs. E2 straw 3 h, *p* = 0.047), likely due to an increase in absolute values of minor defects, which were also statistically comparable except for E2 straw 3 h (*p* = 0.03 vs. fresh and *p* = 0.02 vs. E2 pellet 0 h). Major defects were not affected (*p* = 0.43). Except for what has been just mentioned, there were no differences in morphological anomalies between treatments (extender and system) and post-warming times. Similarly, intact membranes (hypo-osmotic swelling test curly spermatozoa) and intact acrosomes (by FITC-PNA/PI) were significantly higher in fresh samples (*p* = 0.01), but basically showed no differences between ultra-rapid frozen groups, regardless of treatment or time.

Since motility is the preferred parameter to select the spermatozoon to be injected during ICSI, E1 pellet, which showed better maintenance of motility over time, was chosen for Experiment 2.

### Experiment II

After IVM, 97 oocytes resumed meiosis until the MII stage, with 68.79% (97/141) of maturation rate. These oocytes were injected as follows: 32 oocytes were injected with ultra-rapid frozen spermatozoa, 35 oocytes were injected with conventionally slow-frozen spermatozoa, and 30 oocytes were used for sham-ICSI.

Following ICSI, fertilization patterns and embryo development were evaluated. Table 2 shows the distribution of fertilization pattern after oocyte injection with ultra-rapid frozen or conventionally slow-frozen cat spermatozoa. All the normal fertilization patterns (i.e., female pronucleus with decondensed sperm head, two pronuclei, cleavage), as well as the other fertilization patterns (i.e., oocyte activation with intact sperm head, MII arrest with intact sperm head, and MII arrest with decondensed sperm head), were comparable between ultra-rapid frozen and slow-frozen spermatozoa (*p*-values ranging from *p* = 0.075 for MII arrest with intact sperm head to *p* = 1 for oocyte activation with intact sperm head and female pronucleus with decondensed sperm head). The total of normal fertilization patterns, though, tended to be higher in the oocytes injected with control slow-frozen spermatozoa (*p* = 0.05).

**Table 2:** Fertilization patterns of *in vitro* matured feline oocytes after intracytoplasmic sperm injection (ICSI) with conspecific spermatozoa (ultra-rapid frozen or control slow-frozen).

**Spermatozoa**	**Number of injected oocytes**	**Normal fertilization**			**Total normal fertilization**	**Other patterns of fertilization**		
		**Female pronucleus with decondensing sperm head**	**Male and female pronuclei**	**Cleaved embryos**		**Oocyte activation with intact sperm head**	**MII arrest with intact sperm head**	**MII arrest with decondensing sperm head**
		***n*. (%)**	***n*. (%)**	***n*. (%)**	***n*. (%)**	***n*. (%)**	***n*. (%)**	***n*. (%)**
Ultra-rapid frozen	32	1 (3.13)^a^	2 (6.25)^a^	12 (35.5)^a^	15 (46.88)^a^	0 (0)^a^	7 (21.88)^a^	6 (18.75)^a^
Slow-frozen	35	2 (5.71)^a^	4 (11.43)^a^	19 (54.29)^a^	25 (71.43)^b^	0 (0)^a^	2 (5.71)^a^	4 (11.43)^a^

*Classification according to Buarpung et al. (41)*.

*^a, b^Different superscripts indicate significant differences within columns (p ≤ 0.05)*.

*Data from 5 replicates*.

*MII, metaphase II (mature) oocytes*.

**Figure 3:**
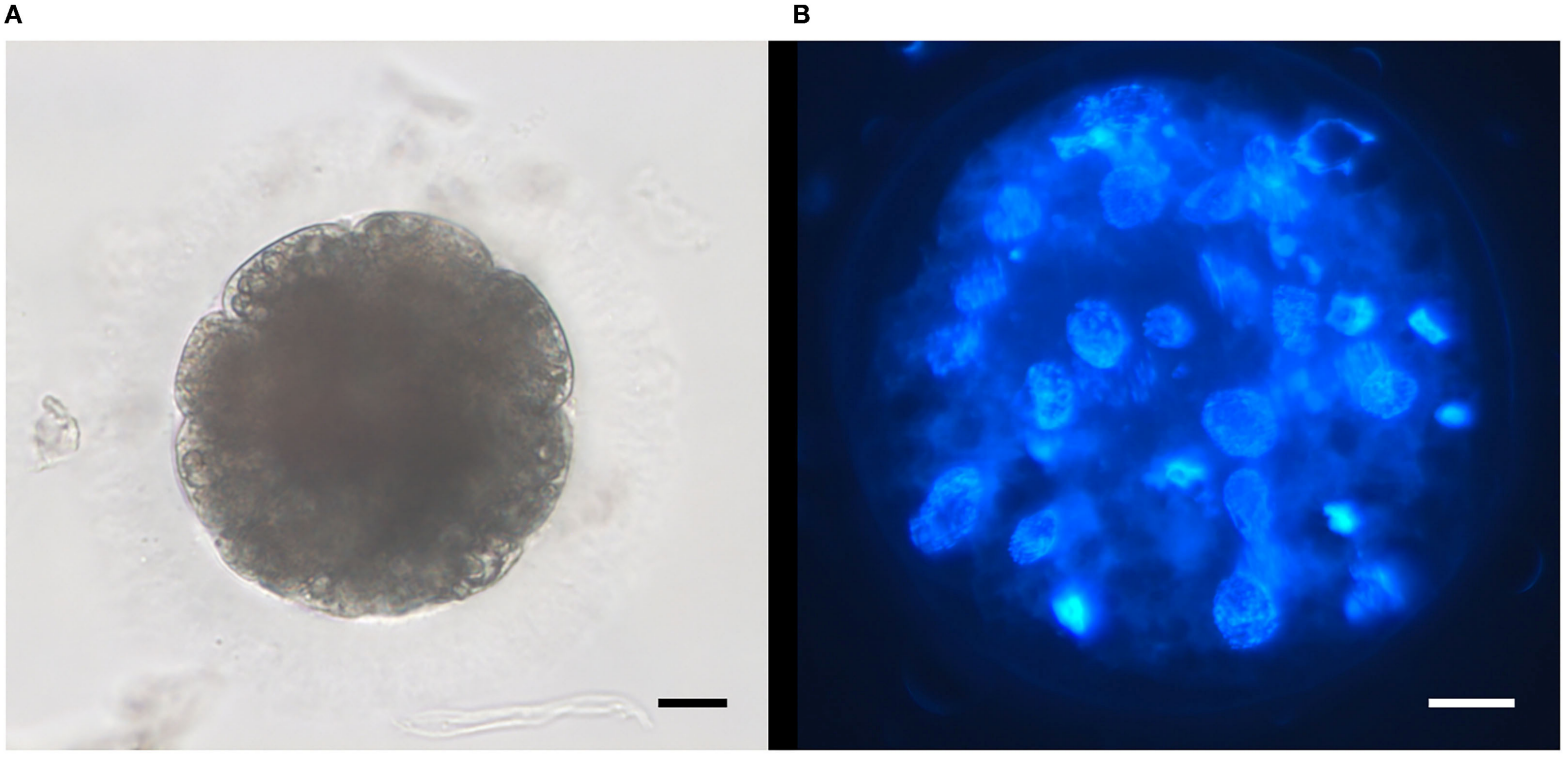
Representative micrographs of embryos obtained after intracytoplasmic sperm injection (ICSI) of ultra-rapid frozen feline spermatozoa. **(A)** Embryo at day 5 of *in vitro* culture; objective 32X. **(B)** Morula after staining with Hoechst at the end of *in vitro* culture (day 7); objective 40X. Scale bars: 20 μm.

Table 3 depicts the progression of embryonic development of cleaved embryos derived from such injected oocytes and their developmental rates. Both ultra-rapid frozen and slow-frozen cat epididymal spermatozoa could successfully fertilize conspecific oocytes and support their embryo development until late stages *in vitro* (morula, Figure 3). No significant differences were found between ultra-rapid frozen and slow-frozen spermatozoa in any developmental stage (*p* = 0.1), confirming the ability of ultra-rapid frozen spermatozoa to produce embryos *in vitro* after ICSI. In sham-injected oocytes, one embryo developed, which was significantly lower than the cleavage of spermatozoa-injected groups (*p* = 0.0012). Degeneration rates were similar among the three groups (ultra-rapid frozen spermatozoa, control slow-frozen spermatozoa, and sham-ICSI, *p* = 0.67).

**Table 3:** Embryonic development of *in vitro* matured feline oocytes after intracytoplasmic sperm injection (ICSI) with conspecific spermatozoa (ultra-rapid frozen or control slow-frozen) or after sham-ICSI.

**ICSI**	**Number of injected oocytes**	**Embryonic development of injected oocytes**				
		**2–4 cells**	**6–8 cells**	**8–16 cells**	**Morulae**	**Degenerated/N.A**.
		***n*. (%)**	***n*. (%)**	***n*. (%)**	***n*. (%)**	***n*. (%)**
Ultra-rapid frozen spermatozoa	32	12 (37.5)^a^	5 (15.63)^a, b^	5 (15.63)^a, b^	4 (12.5)^a^	4 (12.5)^a^
Slow-frozen spermatozoa	35	19 (54.29)^a^	12 (34.29)^a^	9 (25.71)^a^	6 (17.14)^a^	4 (11.43)^a^
Sham	30	1 (3.33)^b^	1 (3.33)^b^	1 (3.33)^b^	1 (3.33)^a^	2 (6.67)^a^

^a, b^*Different superscripts indicate significant differences within columns (p ≤ 0.05)*.

*Data from 5 replicates*.

*N.A., not assessable*.

## Discussion

The application of spermatozoa ultra-rapid freezing, also in the field, would give the chance to exploit such preserved male gametes for the application of assisted reproductive techniques in distant times or places, once conspecific oocytes are available. This work employed two different extenders according to the results of previously published studies. Vizuete et al. (23) assessed the effect of different concentrations of sucrose, a non-permeant cryoprotectant whose protective effect during spermatozoa vitrification was already demonstrated (2, 42). They obtained the best results, especially for what concerns motility, with 0.4 and 0.25 M sucrose combined with 1% BSA (23), and thus, these were the concentrations used for E1 and E2 in this study. Base media, instead, were chosen considering other factors: E1 was based on Tris buffer, which is commonly used for spermatozoa freezing protocols (30, 43), whereas E2 was based on HF-10, which is routinely used for cat epididymal spermatozoa collection and handling in our laboratories, as well as in others (44). In addition, E1 was supplemented with egg yolk, which has the ability to protect the cell membranes thanks to its low-density lipoprotein fraction and is considered an effective agent against thermal shock (1, 45).

Although with a decrease in morphofunctional quality compared to fresh controls, both the extenders used for ultra-rapid freezing in this study resulted in post-warming spermatozoa motility and in the maintenance of some extent of morphological, acrosomal, and membrane integrity. Differences in motility could be due to the different compositions of the extenders. While in (23), a higher concentration of sucrose was beneficial, E1, which only contained 0.25 M sucrose, was the best extender for motility preservation of preserved spermatozoa in this study. The addition of egg yolk in E1 could have been a determinant, and further investigations on the use of egg yolk or phospholipids mixtures during rapid freezing or vitrification are warranted. Cryopreservation, including vitrification and ultra-rapid freezing, causes cells to be exposed to harmful non-physiological conditions. Although spermatozoa should be the easiest cells to cryopreserve, thanks to their small size and large surface/volume ratio (1), different cryopreservation-induced damages have been observed, probably because cat spermatozoa already suffer from a condition of teratozoospermia (26) as evidenced by the percentage of normal morphology in fresh samples. Teratozoospermic samples are more sensitive to osmotic stress (46) and more prone to acrosomal damages (47). In this study, it seemed that the acrosomes were particularly affected by the ultra-rapid freezing procedure. Despite this, acrosome-damaged spermatozoa can still be exploited for embryo production purposes, since the lack of proper acrosome reaction can be overcome by the use of ICSI (48, 49). The results hereby obtained did not differ much from other reports, in which a decline in membrane and acrosome integrity, morphology, and sperm viability is reported in vitrified spermatozoa of different species (23, 50–53). Concerning the ultra-rapid freezing systems, pellets and straws were chosen for their ease of application. Other devices usually employed for oocyte vitrification [Cryotops, (54)] or denudation [stripper tips, (55)] have been tested for spermatozoa vitrification (16, 56), as well as carrier systems specifically designed for this purpose, such as the *SpermVD* (57) or others (58). In this study, pellets and straws were basically comparable, likely because the volume of ultra-rapid frozen spermatozoa was the same in both the systems and the presence of the straw did not have a significant influence on cooling rates. Pellets were chosen for the *in vitro* fertilizing ability assessment of Experiment II because they are easier and faster to produce, they would also be more feasible in field conditions, and they showed some preeminence regarding post-warming motility.

Most likely, the biggest advantage of vitrification and ultra-rapid freezing is that they allow the preservation of small amounts of samples, which could not be frozen. Therefore, they can be considered a useful technique for rescue cryopreservation of male germplasm. Cryopreservation of epididymal spermatozoa from neutered or deceased animals could be the last chance to salvage the genetic material of rare animals or individuals of threatened species, from which other samples will never be obtained. In addition, using pellets for ultra-rapid freezing could also be an asset when dealing with few, precious spermatozoa, because of the possibility to better exploit the preserved material in the future. Sperm cells are preserved in small volumes, and each pellet is single and independent from the others. Compared to slow freezing in straws, this allows to obtain more aliquots from the same sample, and so to optimize sperm doses, which could also be used in distant times. For instance, only one pellet could be warmed for an ICSI, reducing the number of leftover spermatozoa discarded at the end of the procedure and eliminating the need of sperm re-freezing, whose efficiency is controversial in farm species (59, 60) and remains to be investigated in cats. Further developments of spermatozoa cryopreservation could include single sperm vitrification to preserve the fertilizing ability or the genetic material of individuals with severely oligozoospermic samples or reduced number of surgically-retrieved spermatozoa (61).

The use of ICSI as *in vitro* fertilization technique would also be beneficial for male gamete rescue, because of the reduced number of spermatozoa needed, compared to IVF. Its promises for wild animals would be to allow fertilization from suboptimal quality samples, including teratozoospermic samples, which are often collected from males of zoo inbred species (62). In this study, following ultra-rapid freezing, epididymal spermatozoa maintained their fertilizing ability and produced embryos *in vitro* after ICSI. Their fertilizing ability and the capacity to support embryo development were comparable to those of conventionally frozen spermatozoa. In addition, the present results, in terms of fertilization success, were also similar to those reported in the literature employing ejaculated spermatozoa, of higher quality, after vitrification and IVF (8). Therefore, ultra-rapid freezing of epididymal spermatozoa could be an alternative to conventional freezing, and its combination with ICSI would allow its wide application for a variety of male samples and situations. Its investigation on other species could be a contribution to animal gamete biobanks and could support biodiversity preservation altogether.

However, before vitrification or ultra-rapid freezing can be used as the standard cryopreservation protocols, further optimization and *in vivo* studies are granted. Sperm parameters, especially motility, should be improved to increase the number of usable gametes and speed up the ICSI procedure. Besides, ICSI itself should also be ameliorated. What was hereby classified as “other patterns of fertilization” could be due to either the oocyte or the spermatozoon, as evidenced by metaphase II arrest or lack of spermatozoa head decondensation. It is still unknown whether the use of cryopreserved sperm cells for ICSI could increase the amount of un-decondensed heads, but this finding was already reported after ICSI of spermatozoa derived from frozen testicular tissue (41). Molecular investigation on ultra-rapid frozen spermatozoa would be necessary to assess the specific mechanisms leading to these fertilization patterns, including the study of the cell membrane, which could be affected by the cryopreservation and influence the sperm capacity to release its genetic material (63, 64), as well as DNA fragmentation, which could hinder spermatozoa fertilizing ability, and the effect of ultra-rapid freezing on “Sperm factors” (e.g., phospholipase C zeta (PLCζ), a candidate sperm-derived oocyte-activating factor). On the other hand, the block of oocyte activation and progression of fertilization events could partially be overcome by artificial oocyte activation, which, however, was not applied in this study to avoid potential parthenogenetic activation instead of normal fertilization (65). Only a joint effort to improve both cryopreservation and fertilization outcomes will lead to a truly efficient strategy for the rescue of male gametes from single individuals with poor semen characteristics.

In conclusion, extender 1 (Tris-egg yolk + 0.25*M* sucrose) better maintained the morphofunctional integrity of cat epididymal spermatozoa following ultra-rapid freezing, and the use of pellets allowed to maintain the fertilizing ability of such preserved spermatozoa, which could produce embryos after ICSI. Therefore, ultra-rapid freezing in pellets represents a viable possibility for a simple field cryopreservation procedure of male germplasm. With further optimization, this method could increase the possible locations for spermatozoa preservation, could allow multiple uses of the same batch of preserved cells, one pellet at the time, and could foster the creation of feline gamete biobanks.

## Data Availability Statement

The raw data supporting the conclusions of this article will be made available by the authors, without undue reservation.

## Ethics Statement

Ethical review and approval was not required for the animal study because it used domestic cat gonads collected as byproducts from routine surgeries.

## Author Contributions

MC and JZ: conceptualization and methodology. MC and MM: investigation. MC: data curation, formal analysis, and writing—original draft. MC, MM, JZ, and GL: writing—review and editing. GL: conceptualization, resources, funding acquisition, and project administration. All authors read and approved the submitted version of the article.

## Funding

This article has been supported by the Polish National Agency for Academic Exchange under Grant No. PPI/APM/2019/1/00044/U/00001 and the authors acknowledge support from the University of Milan through the APC initiative.

## Conflict of Interest

The authors declare that the research was conducted in the absence of any commercial or financial relationships that could be construed as a potential conflict of interest.

## Publisher's Note

All claims expressed in this article are solely those of the authors and do not necessarily represent those of their affiliated organizations, or those of the publisher, the editors and the reviewers. Any product that may be evaluated in this article, or claim that may be made by its manufacturer, is not guaranteed or endorsed by the publisher.
